# Unraveling Benign Breast Conditions: A Comprehensive Study of Diagnosis, Treatment, and Care

**DOI:** 10.7759/cureus.85183

**Published:** 2025-06-01

**Authors:** Priya Ahire, Pooja Nagwani, Rukmini Waghmare, Ami Gandhi

**Affiliations:** 1 General Surgery, Grant Government Medical College and Sir JJ Group of Hospitals, Mumbai, IND; 2 General Surgery, Grant Government Medical College, Mumbai, IND

**Keywords:** benign breast condition, breast fibroadenoma, fibrocystic breast changes, lactational mastitis, lumpectomy, lump in breast

## Abstract

Introduction

The breast undergoes cyclical changes influenced by hormones, with benign breast diseases (BBDs) being more common than malignant conditions. These conditions can present with symptoms such as lumps, pain, swelling, and nipple discharge. This study investigates the incidence, clinical features, age distribution, and management of BBDs, providing insight into the common breast-related issues that affect women across different stages of life.

Methodology

A prospective observational study was conducted at a tertiary care hospital from August 2021 to December 2022, including 100 patients with breast-related complaints. Participants underwent detailed history, clinical examination, and relevant investigations such as ultrasonography, mammography, and fine needle aspiration cytology (FNAC). Data were analyzed using Excel and SPSS, with continuous variables presented as mean ± standard deviation and categorical data as numbers and percentages.

Results

In the present study, the mean age of the 100 participants with BBD was 34.98±11.97 years, with the majority (78/100, 78%) presenting with lumps. Fibroadenoma was the most common diagnosis, seen in 59/100 (59%), followed by fibroadenosis in 19/100 (19%) and breast abscess in 16/100 (16%). The mean age of menarche among patients with BBD was 14.52±1.80 years, with 56/100 (56%) attaining menarche between 13 and 15 years. Additionally, 81/100 (81%) were married, and 10/100 (10%) had a history of lactation. The majority of patients with BBD had lumps in the right upper outer quadrant, with most lumps being firm and mobile, and 97/100 (97%) showed no axillary lymph node involvement. Most patients were treated with excision, while others received conservative treatment with reassurance, analgesics, and antibiotics, with diagnosis confirmed through ultrasonography and mammography.

Discussion

The spectrum of BBDs in our study aligns with findings from other studies, with fibroadenoma being the most prevalent lesion, followed by fibrocystic changes. These conditions predominantly affect young women under the age of 40 in our population.

Conclusion

In conclusion, BBDs, particularly fibroadenoma, are common and require accurate clinical assessment, with FNAC being a reliable diagnostic tool. Early detection through breast self-examination and regular follow-ups, along with continued education on benign breast conditions, is essential for effective management and patient care.

## Introduction

The breast is a complex gland and is a dynamic structure because it undergoes changes throughout life in the composition of cell and gene expression [1]. The breast undergoes cyclical changes throughout reproductive life, influenced by hormones during puberty, menstruation, and menopause. Benign breast disease (BBD) is highly prevalent among women of reproductive age and although BBD is an established risk factor for breast cancer, the degree of association varies depending on the specific type of lesion [2]. BBDs represent a spectrum of disorders that range from extreme normality to well-defined disease processes. It includes cyclical mastalgia, physiological swelling, palpable lumps, nipple discharge, infection, or inflammation. It is the most common cause of breast problems in females and is more frequent than malignant diseases [3-5]. When evaluating a lump, surgeons must determine if it is abnormal and rule out malignancy to ease the patient’s anxiety. Cytological analysis is commonly used to diagnose breast lumps. Fine needle aspiration cytology (FNAC) with immediate reporting offers early reassurance and helps avoid unnecessary surgery while identifying breast cancer cases. Historically, benign breast disorders were undervalued, with unclear terminology and poor correlation between clinical, radiological, and pathological findings [6]. The popular classification of BBDs is according to the Aberration of the Normal Development and Involution (ANDI), which is used to describe the spectrum of BBD [7]. This study (N=100) aims to evaluate the incidence, clinical presentation, age distribution, and management of BBDs, providing insight into the common breast-related issues that affect women across different stages of life.

## Materials and methods

A prospective observational design was conducted at the Department of General Surgery in a tertiary care teaching hospital. It included patients presenting with breast-related complaints in both the outpatient and inpatient departments from August 2021 to December 2022, covering an 18-month period. A total of 100 patients with breast-related complaints, who met the eligibility criteria, were included in the study. The study was approved by the institutional ethical committee, and all enrolled patients (N=100) were briefed about the study and provided written informed consent.

Inclusion criteria

The inclusion criteria consisted of patients diagnosed with BBDs, aged between 12 and 80 years, including both males and females, and those willing to participate in the study with written informed consent.

Exclusion criteria

Patients were excluded if they had an obvious malignant disease, had previously been treated for malignancy, had congenital anomalies, or were unwilling to participate in the study.

Methodology

Each patient underwent a detailed history, clinical examination, and relevant investigations including ultrasonography of the breast and axilla, mammography, FNAC, and treatment.

Statistical analysis

For statistical analysis, data entry and analysis were performed using Excel and SPSS (ver. 27, IBM Co., New York, NY, USA). Continuous data were presented as mean ± standard deviation, while categorical data were expressed as numbers and percentages in tables and figures.

## Results

**Figure 1:**
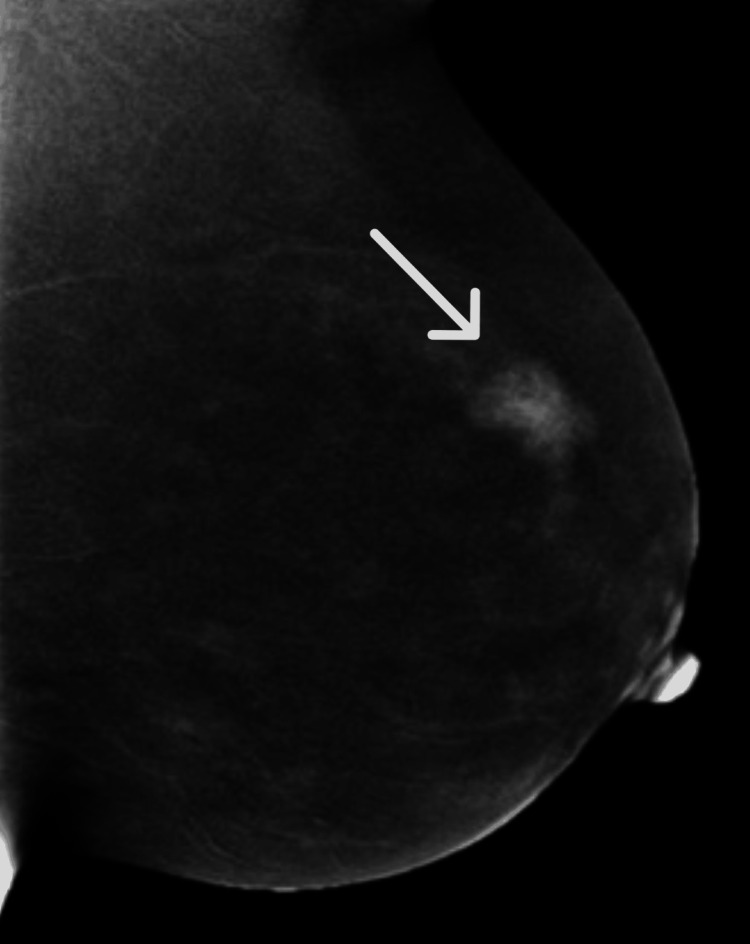
Mammographic image of breast fibroadenoma (white arrow).

In our study of 100 participants with BBD, the mean age was 34.98±11.97 years. The majority of patients were in the 21-40 age group. Among the participants, 78 (78%) presented with a breast lump, followed by 37 (37%) with pain, 16 (16%) with nodularity, seven (7%) with fever, and five (5%) with nipple discharge. Regarding diagnosis, 59 (59%) of the patients had fibroadenoma, followed by fibroadenosis in 19 (19%), breast abscess in 16 (16%), mastitis in three (3%), fibrocystic disease in two (2%), and phyllodes tumor in 1 (1%) patient, as shown in Table 1. Figure 1 displays a mammographic image of breast fibroadenoma.

**Table 1:** Distribution of benign breast disease among the participants (N=100).

Diagnosis	Frequency (%)
Fibroadenoma	59 (59%)
Fibroadenosis	19 (19%)
Mastitis	3 (3%)
Abscess	16 (16%)
Phyllodes	1 (1%)
Fibrocystic	2 (2%)

**Figure 2:**
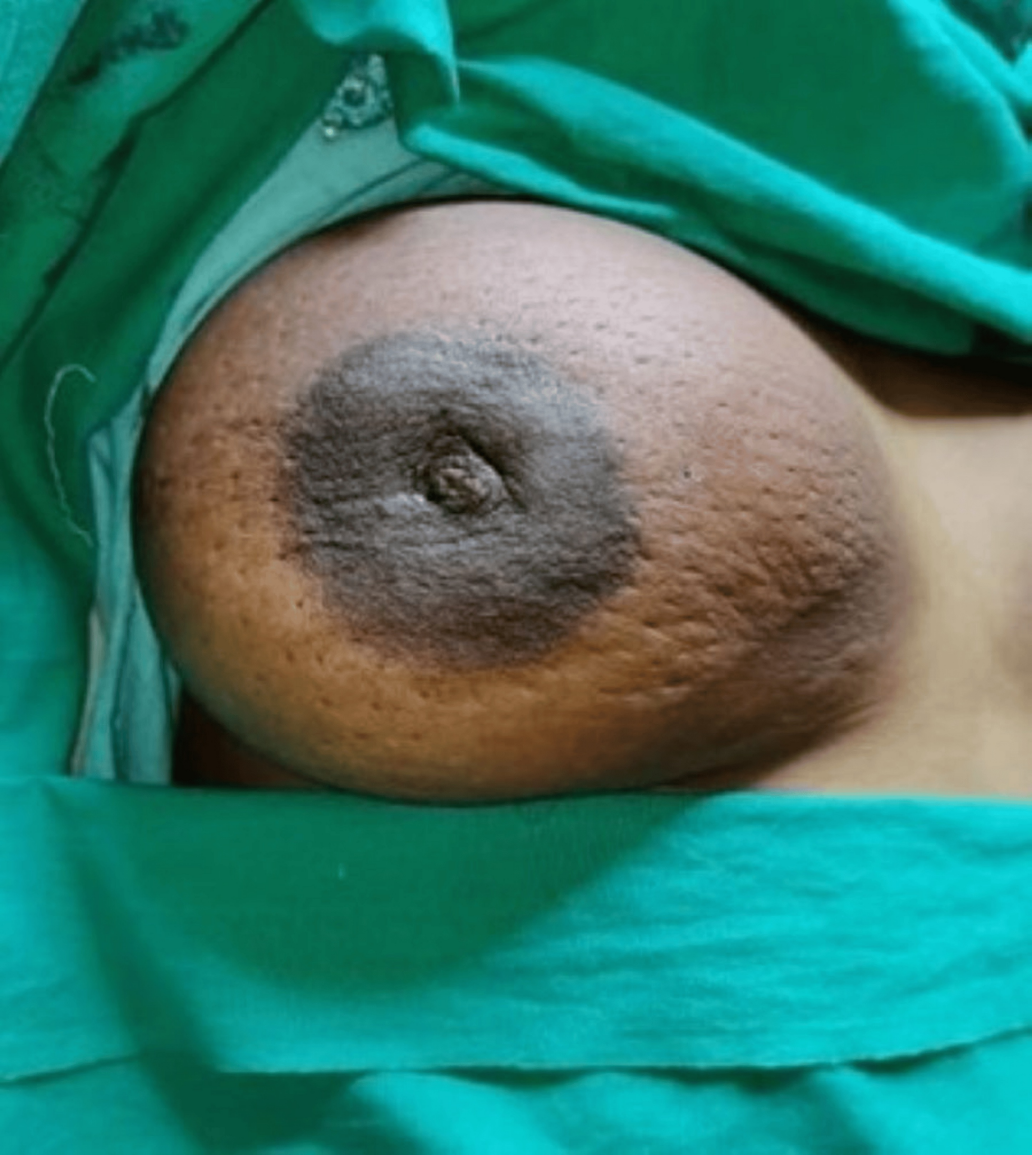
Engorged right breast showing a lactational breast abscess.

In evaluating the risk factors among 100 patients with BBD, the mean age of menarche was 14.52±1.80 years. A total of 81 (81%) participants were married, one (1%) had a history of the oral contraceptive pill (OCP) use, and 10 (10%) had a history of lactation. Figure 2 shows a clinical image of a lactational breast abscess. Among the participants, the majority, 56 (56%), attained menarche between the ages of 13 and 15, followed by 33 (33%) between 16 and 18 years, and 11 (11%) between 10 and 12 years.

**Figure 3:**
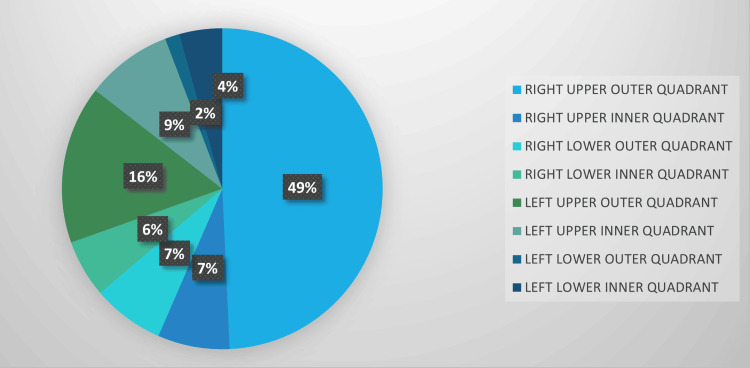
Pie chart depicting the site of the lump among study subjects (n=100).

On evaluating the characteristics of the lump among 100 patients, 34 (34%) had breast lumps in the right upper outer quadrant, followed by the left upper outer quadrant. Most patients had right breast involvement. The majority of lumps were firm in consistency, and 62 (62%) were mobile. In most patients, the skin over the breast was red and warm. The axillary lymph node was not palpable in 97 (97%) of the patients. Table 2 shows the characteristics of BBD among the study subjects. Figure 3 illustrates the quadrant-wise distribution of the breast lumps.

**Table 2:** Characteristics of the benign breast disease among the study subjects (N=100).

Parameter	Category	Frequency (%)
Consistency	Cystic	15 (15%)
	Firm	63 (63%)
	Indurated	1 (1%)
Total		100 (100%)
Mobility	Mobile	62 (62%)
	Not mobile	38 (38%)
Total		100 (100%)
Nipple discharge	Present	4 (4%)
	Absent	96 (96%)
Total		100 (100%)
Skin over breast	Red, indurated	1 (1%)
	Red, tender	3 (3%)
	Red, warm	5 (5%)
	Red, tender, warm	3 (3%)
	Red, tender, warm, engorged vein	1 (1%)
	Gangrenous	1 (1%)
Total		100 (100%)
Axillary lymph nodes	Palpable	3 (3%)
	Not palpable	97 (97%)
Total		100 (100%)

**Figure 4:**
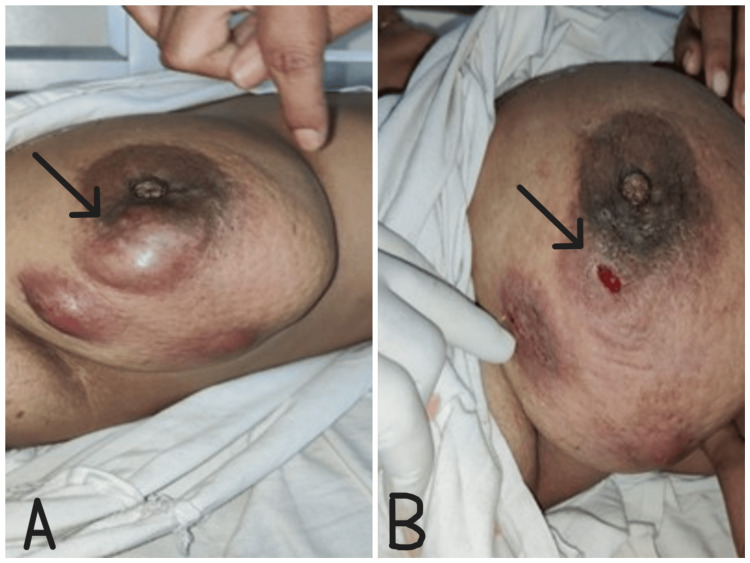
A: Clinical image of right-sided granulomatous mastitis (black arrow). B: Post-procedure clinical image of the right breast after incision and drainage (black arrow).

On comparing diagnosis with lump size, the majority of patients with BBD had breast lumps measuring 2-5 cm. Diagnosis was made using ultrasonography and mammography according to clinical indications. In our study, most patients with BBD were treated with excision, followed by conservative treatment including reassurance, analgesics, and antibiotics, as shown in Table 3. Figure 4 shows clinical images of granulomatous mastitis of the right breast before and after the procedure.

**Table 3:** Management of benign breast diseases encountered in the present study (N=100).

Diagnosis	Aspiration (%)	Excision (%)	Incision and drainage (%)	Conservative (%)	Wide local excision (%)
Fibroadenoma	-	49 (49%)	-	10 (10%)	-
Fibroadenosis	-	-	-	19 (19%)	-
Mastitis	-	-	1 (%)	2 (2%)	-
Abscess	6 (6%)	-	7 (7%)	3 (3%)	-
Phyllodes	-	-	-	-	1 (1%)
Fibrocystic	-	-	-	2 (2%)	-

## Discussion

Patients with BBDs typically present with symptoms such as breast lumps, pain, or nipple discharge. The ANDI classification, introduced by an international working group in 1992, categorizes them based on pathogenesis and the degree of abnormality, recognizing that most BBDs stem from normal physiological processes [7]. However, despite its solid pathophysiological foundation, it has not been widely used in epidemiologic studies or clinical practice, with most clinicians opting for separate clinical and pathological classifications. To ensure early diagnosis, patients presenting with a combination of symptoms should undergo a thorough history review followed by a triple assessment.

Age

In our study, the mean age among the patients with BBD was 34.98±11.97. Most patients were in the age group of 21-40. Similarly, Londen SJ et al. [8] and LaVecchia C et al. [9] in their study reported that the incidence of BBDs begins to rise in the second decade, and it peaks in the fourth or fifth decades compared to the malignant lesions, for which the incidence continues to increase after menopause. In concurrence with our study, Kumar et al. [10] reported that most patients with BBD were aged 21-30, followed by 31-40, with a mean age of presentation of 29.3 years.

Most patients in this study presented late, mainly due to financial constraints and painless lumps. Other factors included the hope that the lump would disappear, delayed referrals, lack of interference with daily activities, and lack of awareness. The delay in seeking care for breast lumps, compared to breast pain, highlights the need for better health education on breast cancer risk factors.

Symptoms

In our study, 78% of patients presented with lumps, followed by 37% with pain, 16% with nodularity, 7% with fever, and 5% with discharge. Navneet Kaur et al. reported that 68.3% of patients had lumps, followed by mastalgia with nodularity. Mima Maychet et al. found that 87% of patients with BBD had breast lumps, while Foncroft LM et al. reported 87.4% presented with lumps. Similarly, Kumar et al. [10] reported that 88% of patients presented with breast lumps, followed by 37.3% with cyclical breast pain and 8.6% with nipple discharge.

Site of benign breast disease

In the current study analyzing the site of BBD, the most common location was the right upper quadrant. Similarly, in a study by Mallikarjuna et al. [11], the most frequently involved quadrants were the upper outer, lower outer, upper inner, and lower inner quadrants. Likewise, Adesunkami Agbakwuru et al. [12] reported that the most common anatomical site of the breast lump was the upper outer quadrant.

Diagnosis

In our study, the majority of patients (51%) were diagnosed with fibroadenoma, followed by fibroadenosis in 19%, breast abscess in 16%, fibrocystic disease in 10%, mastitis in 3%, and phyllodes tumor in 1%. In a study by Kumar et al. [10], fibroadenoma (42%) was the most common diagnosis among patients with BBD, followed by fibroadenosis (16%) and breast abscess (13%). Maychet MB et al. [9] reported that fibroadenomas accounted for 87% of benign breast lumps. Similarly, studies by Adesunkanmi AR et al. [12], Greenberg R et al. [13], Ihekwaba FN et al. [14], and Florica JV et al. [15] also found that fibroadenoma was the most common benign breast disease, with its frequency ranging from 46.6% to 55.6%.

Risk factors

Kelsey JL et al. [16], Dupont WD et al. [17], and London SJ et al. [18] found that early menarche, short menstrual cycles, nulliparity, older age at first birth, OCP use, hormone replacement therapy (HRT), and high postmenopausal BMI increase the risk of breast cancer, while longer durations of breastfeeding and higher premenopausal BMI reduce it. In our study, the mean age of menarche was 14.52±1.80 years; 81% of participants were married, 1% had a history of OCP use, and 10% were lactating. Goehring C et al. [19] and Wang DY et al. [20] reported that OCP use is protective against BBDs, while HRT increases the risk. They also found no association between age at menarche and the risk of BBD, aligning with our findings.

Management

In our study, most patients underwent surgical intervention, with lumpectomy being the most common procedure, followed by conservative treatment. This pattern is consistent with findings reported in other studies in the literature. The spectrum of BBDs in our study population does not significantly differ from other studies, with fibroadenoma being the most common benign breast lesion, followed by fibrocystic changes. BBDs in this population primarily affect young women under 40 years of age.

Limitations

The present study had a relatively small sample size, which may limit the generalizability of the findings. Additionally, the lack of long-term follow-up restricts the ability to assess recurrence or complications. Future studies should include larger and more diverse samples, along with long-term follow-up, to better evaluate recurrence and complications. Multivariate analyses are also needed to improve treatment approaches and refine management strategies for BBD.

## Conclusions

In conclusion, BBDs represent the majority of breast-related conditions, with fibroadenoma being the most common benign lesion. This study highlights the importance of accurate clinical assessment, with FNAC proving to be a reliable diagnostic tool, particularly for fibroadenomas. The conservative management approach is acceptable in adolescent patients but requires careful evaluation and informed decision-making. In adult women, a triple-test approach remains essential before considering conservative management. Early detection through breast self-examination and regular clinical follow-ups is crucial to ensuring optimal patient care. Thus there is the need for continued education and awareness regarding benign breast conditions, especially for promoting early detection and management strategies.
